# Is visceral leishmaniasis a sepsis or not?

**DOI:** 10.1186/cc11802

**Published:** 2012-11-14

**Authors:** E Puca, P Pipero, P Pilaca, E Puca

**Affiliations:** 1 University Hospital Center 'Mother Teresa', Tirana, Albania; 2 American Hospital, Tirana, Albania

## Background

Based on a sepsis consensus conference in 1992 and after that in 2001, sepsis is defined as a syndrome by the presence of both infection and a systemic inflammatory response (SIRS). SIRS is considered to be present when patients have more than one of the following clinical findings: body temperature >38°C or <36°C; heart rate >90/minute; hyperventilation evidenced by a respiratory rate >20/minute or PaCO_2 _<32 mmHg; and white blood cell count >12,000 cells/μl or <4,000/μl [1]. On the other hand visceral leishmaniasis (VL), or kala-azar, is a parasitic disease that consists of a protracted course of fever, pallor, wasting, hepatosplenomegaly and pancytopenia [2,3]. The aim of our study is to start a discussion about VL and sepsis.

## Methods

From 45 adults patients diagnosed with VL between January 2005 and December 2009 in the Service of Infectious Diseases, University Hospital Centre of Tirana, Albania, we have selected 36 patients who presented with fever >38°C and leucopenia <4,000/μl. The diagnosis of VL was based on demonstration of leishmania parasites in bone marrow smears.

## Results

From 36 patients 58.3% were males and 41.6 were females. Age ranged from 17 to 69 years, the average age was 43.2 years. Main clinical and laboratory findings were: fever in 100%, malaise in 100%, hepatosplenomegaly in 100%, anemia in 82%, leucopenia in 100%, thrombocytopenia in 50%, and increased liver enzymes in 52.7% of cases. Bone marrow aspirate was performed in all cases with amastigotes identified in 100% of the cases. Meglumine antimoniate was used in all cases as an initial treatment. Treatment failure occurred in two cases (5.5%) that were treated subsequently with liposomal amphotericin B. The case fatality was 5.5%. The main causes of death were liver and cardiac failure.

## Conclusion

Based on the clinical and laboratory findings VL could be considered a systemic inflammatory response. Maybe in this disease we have not evaluated an inflammatory test like C-reactive protein or procalcitonin to be convicted if higher than the normal value, but we think we need to start a study about markers of sepsis in this systemic parasitic disease, because our opinion is that VL is a SIRS plus parasitic systemic infection - which does mean sepsis. In malaria, PCT levels are elevated in both severe and uncomplicated *Plasmodium falciparum *malaria, but how useful is this inflammatory test in VL [4]?
